# A Novel Insight into the Cardiotoxicity of Antineoplastic Drug Doxorubicin

**DOI:** 10.3390/ijms141121629

**Published:** 2013-10-31

**Authors:** Zbynek Heger, Natalia Cernei, Jiri Kudr, Jaromir Gumulec, Iva Blazkova, Ondrej Zitka, Tomas Eckschlager, Marie Stiborova, Vojtech Adam, Rene Kizek

**Affiliations:** 1 Department of Chemistry and Biochemistry, Faculty of Agronomy, Mendel University in Brno, Zemedelska 1, Brno CZ-613 00, Czech Republic, E-Mails: heger@mendelu.cz (Z.H.); cernei.natalia3@gmail.com (N.C.); george.kudr@centrum.cz (J.K.); j.gumulec@gmail.com (J.G.); iva.blazkova@seznam.cz (I.B.); ZitkaO@seznam.cz (O.Z.); vojtech.adam@mendelu.cz (V.A.); 2 Central European Institute of Technology, Brno University of Technology, Technicka 3058/10, Brno CZ-616 00, Czech Republic; 3 Department of Pathological Physiology, Faculty of Medicine, Masaryk University, Komenskeho namesti 2, Brno CZ-662 43, Czech Republic; 4 Department of Paediatric Haematology and Oncology, 2nd Faculty of Medicine, Charles University, and University Hospital Motol, V Uvalu 84, Prague 5 CZ-15006, Czech Republic; E-Mail: Tomas.Eckschlager@fnmotol.cz; 5 Department of Biochemistry, Faculty of Science, Charles University, Albertov 2030, Prague 2 CZ-12840, Czech Republic; E-Mail: stiborov@natur.cuni.cz

**Keywords:** myocardium, cardiomyopathy, interaction, amide bond, spectrophotometry, ion-exchange liquid chromatography

## Abstract

Doxorubicin is a commonly used antineoplastic agent in the treatment of many types of cancer. Little is known about the interactions of doxorubicin with cardiac biomolecules. Serious cardiotoxicity including dilated cardiomyopathy often resulting in a fatal congestive heart failure may occur as a consequence of chemotherapy with doxorubicin. The purpose of this study was to determine the effect of exposure to doxorubicin on the changes in major amino acids in tissue of cardiac muscle (proline, taurine, glutamic acid, arginine, aspartic acid, leucine, glycine, valine, alanine, isoleucine, threonine, lysine and serine). An *in vitro* interaction study was performed as a comparison of amino acid profiles in heart tissue before and after application of doxorubicin. We found that doxorubicin directly influences myocardial amino acid representation even at low concentrations. In addition, we performed an interaction study that resulted in the determination of breaking points for each of analyzed amino acids. Lysine, arginine, β-alanine, valine and serine were determined as the most sensitive amino acids. Additionally we compared amino acid profiles of myocardium before and after exposure to doxorubicin. The amount of amino acids after interaction with doxorubicin was significantly reduced (*p* = 0.05). This fact points at an ability of doxorubicin to induce changes in quantitative composition of amino acids in myocardium. Moreover, this confirms that the interactions between doxorubicin and amino acids may act as another factor most likely responsible for adverse effects of doxorubicin on myocardium.

## 1. Introduction

Doxorubicin, an anthracycline antibiotic, is widely used to treat a number of cancers [1–3], including as breast and lung cancers [4–6]. The first anthracyclines were isolated from the pigment-producing *Streptomyces peucetius* var. *caesius* in the 1960s and named doxorubicin (DOX) and daunorubicin (DNR) [7]. They still remain one of the most effective chemotherapeutic antitumor agents [8], which effect is based on intercalation into DNA helix [9]. In addition, they inhibit the activity of enzyme topoisomerase II that prevents DNA repairing [10–12]. Doxorubicin (DOX) also acts by stabilizing a reaction, in which DNA strands are cut and covalently connected to the tyrosine residues of topoisomerase II, eventually impeding DNA resealing. Topoisomerase II-induced DNA damage is followed by a growth arrest in the G_1_ and G_2_ phases and apoptosis [13]. Anthracyclines exert their cytotoxic effect also by generating reactive oxygen species (ROS) [14], such as H_2_O_2_ and superoxide anion radical [15]. These pro-oxidant properties of DOX have a potential to induce cell death through an oxidative damage of mitochondria [16].

On the other hand, doxorubicin may cause several side effects [17,18], which are mainly evidenced by serious deteriorations of the cardiac muscle including dilated cardiomyopathy [19–21], congestive heart failure [22,23], arrhythmias [24] and also myelotoxicity [7]. All these effects significantly limit clinical use of DOX. Despite the extensive studies of the cardiotoxicity of DOX at cellular, biochemical, molecular, and genetic levels, it has not satisfactorily been elucidated yet [7,11]. Most likely, it is a multifactorial process where alterations in cellular structure [25], formation of ROS that attack the non-target structures [26,27], and induction of apoptosis [20,28] play the important roles. Therefore, new strategies for decreasing the cardiotoxicity of DOX are looking for.

Amino acids exert a cardioprotective effect in ischemia and other cardiac disorders. They play a crucial role in the cardiac metabolism as a source of acetyl-CoA, and contribute to the production of NADH and FADH_2_ and conversion of glutamine and glutamate to free radical scavengers [29,30]. However, the relevance of amino acid metabolism in the general population suffering from heart diseases remains still poorly elucidated [31].

The *in vitro* ion-exchange liquid chromatographic (IELC) and spectrophotometric studies of interactions between fundamental amino acids contained in myocardium with major representative of the anthracycline cytostatics doxorubicin and comparison of content and representation of amino acids in myocardium before and after exposure to doxorubicin were the most important aims of this study. We also determined the breaking points, the critical amount of DOX that is sufficient for formation of mutual complexes for each amino acid.

## 2. Results and Discussion

### 2.1. Amino Acid Profile of Chicken Myocardium

**Figure 1:**
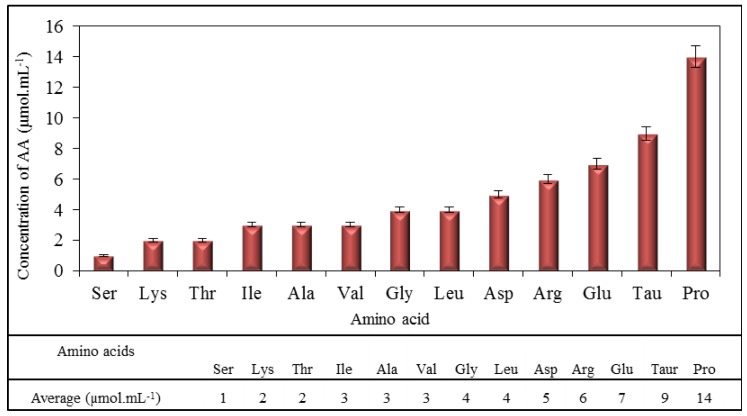
Average content of amino acids in chicken hearts (average of measurements of 10 samples). Measurements were carried out using ion-exchange liquid chromatography (IELC) with postcolumn derivatization with ninhydrin.

The purpose of the study was to investigate the influence of DOX on major amino acids present in myocardium. Proline, taurine, glutamic acid, arginine, aspartic acid, leucine, glycine, valine, alanine, isoleucine, threonine, lysine and serine in the downward trend were determined as the most common amino acids in the amino acid profile obtained by IELC. Their concentrations ranged from 1 μmol mL^−1^ for serine to 14 μmol mL^−1^ for proline (Figure 1), where values of concentrations were obtained as the averages from ten independent measurements. These values ranged in amounts similar to values of some amino acids that were determined in human heart in the study by Weitzel *et al.* [32]. All these amino acids were subsequently used for monitoring possible interactions with DOX.

### 2.2. Spectrophotometric Analysis of Amino Acids-Doxorubicin Interactions

**Figure 2:**
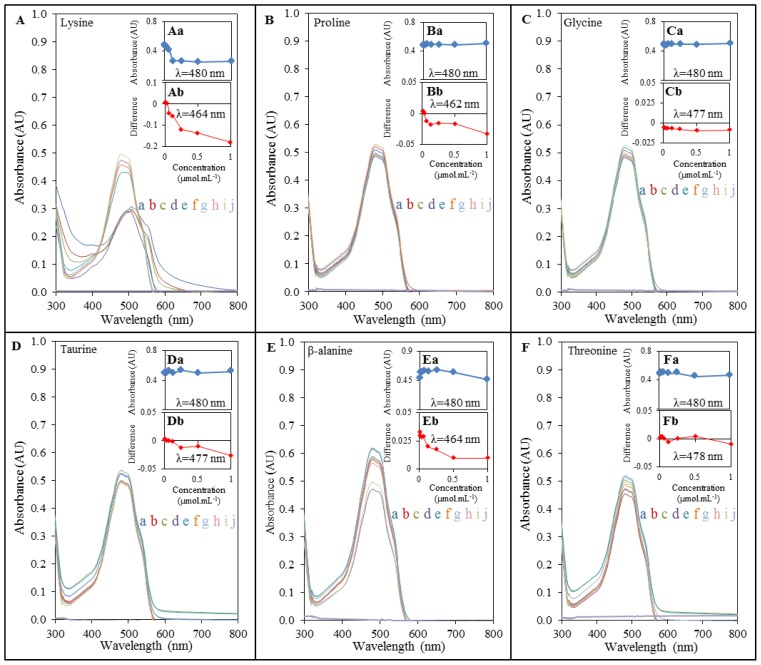
Interactions of amino acids (1; 2; 3; 6; 12; 25; 50; 100 μmol mL^−1^) with doxorubicin (100 μg mL^−1^) monitored with spectrophotometry. DOX interaction with: (**A**) lysine; (**B**) proline; (**C**) glycine; (**D**) taurine; (**E**) β-alanine, (**F**) threonine; where (**a**) stays for 100 μmol mL^−1^; (**b**) 50 μmol mL^−1^; (**c**) 25 μmol mL^−1^; (**d**) 12 μmol mL^−1^; (**e**) 6 μmol mL^−1^; (**f**) 3 μmol mL^−1^; (**g**) 2 μmol mL^−1^; (**h**) 1 μmol mL^−1^; (**i**) 0 μmol mL^−1^ and (**j**) for control (AA without DOX). The dependence of the DOX absorbance at 480 nm on the different concentrations of amino acids is shown in insets marked with lowercase letter a. Insets marked with lowercase letter b express the differences obtained from differential spectra gained as a readout of DOX spectrum from DOX-AA complex spectrum with observed wavelength maximum changes.

**Figure 3:**
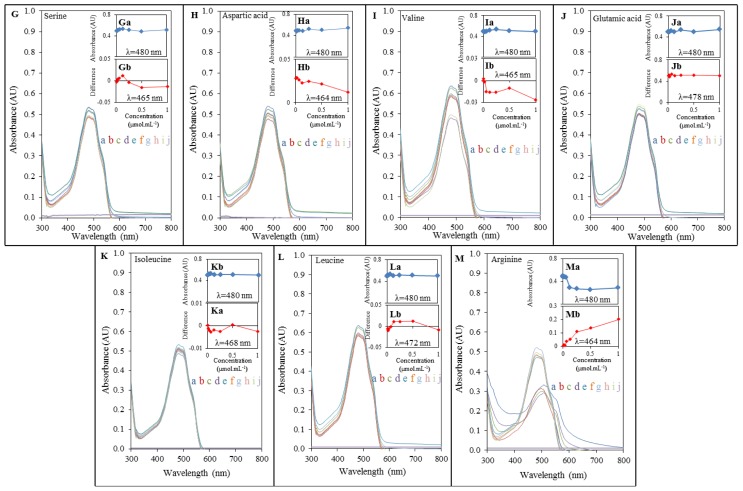
Interactions of amino acids (1; 2; 3; 6; 12; 25; 50; 100 μmol mL^−1^) with doxorubicin (100 μg mL^−1^) monitored with spectrophotometry. DOX interaction with: (**G**) serine; (**H**) aspartic acid; (**I**) valine; (**J**) glutamic acid; (**K**) isoleucine; (**L**) leucine; (**M**) arginine, where (**a**) stays for 100 μmol mL^−1^; (**b**) for 50 μmol mL^−1^; (**c**) 25 μmol mL^−1^; (**d**) 12 μmol mL^−1^; (**e**) 6 μmol mL^−1^; (**f**) 3 μmol mL^−1^; (**g**) 2 μmol mL^−1^; (**h**) 1 μmol mL^−1^; (**i**) 0 μmol mL^−1^ and (**j**) for control (AA without DOX). The dependence of the absorbance at 480 nm on the different concentrations of amino acids can be observed in insets marked with lowercase letter a. Insets marked with lowercase letter b express the differences obtained from differential spectra gained as readout of DOX spectrum from DOX-AA complex spectrum with observed wavelength changes.

To investigate DOX-induced interactions with amino acids, we primarily used an UV-VIS spectrophotometric method. Marked interactions of lysine, β-alanine, valine, and arginine respectively were evident from the obtained spectra. The spectra of interactions of DOX with other amino acids pointed at no interactions at the constant concentration of doxorubicin when compared different concentrations of AA (Figures 2 and 3). Doxorubicin exhibited the maximum at λ = 480 nm, which corresponds to findings published in several studies [33–35]. Lysine (Figure 2A), whose amino group is highly reactive and often participates in enzymatic reactions [29], β-alanine (Figure 2E), serine (Figure 3G), valine (Figure 3I) and arginine (Figure 3M) showed significantly stronger interaction with DOX with the increasing concentration (in a concentration dependent manner; highest effects observed at the concentration of 1000 μg mL^−1^) and thus indicated the highest binding affinity for the formation of complex with DOX. This phenomenon was monitored up to the concentration of 3 μmol mL^−1^ (serine), and 12 μmol mL^−1^ (lysine, arginine, β-alanine, valine and aspartic acid). Other amino acids showed interactions with DOX in much higher concentrations applied as it is apparent from doxorubicin peaks. Trends confirming the above mentioned facts can be seen in insets (a) in Figures 2 and 3, which indicate relations between the concentration of amino acid interacting with DOX and its subsequent effect on absorbance of DOX. Decreasing trend in the series serine, lysine, β-alanine, valine, and arginine points at higher interaction rates and at a higher content of individual amino acids. The insert curves showed no significant changes in the case of all other amino acids.

In addition, we also processed differential spectra of DOX-AA interactions. These results show real forms of interaction output and its real wavelength (insets (a) in Figures 2 and 3). When doxorubicin subjected to interaction with serine, lysine, arginine, β-alanine, valine and aspartic acid, shifts of maximum wavelengths within the range from 464 to 465 nm were observed. Similar wavelength shift was observed also in the case of proline that changed wavelength of doxorubicin to λ = 462 nm. Other amino acids exhibited relatively small shifts of DOX wavelength, but the differences were present at all of them. These changes mention the amendment to structural changes of molecule of DOX, which is capable to form a complex with amino acid. Curves in insets (a) in Figures 2 and 3 point at weak interaction between DOX, proline and glycine. This interaction is not influenced by concentrations of these amino acids. Low effects of AA concentrations were also observed in the case of leucine, isoleucine, and threonine. Interestingly, among these amino acids the smallest wavelength changes of doxorubicin were also observed. These amino acids were shown as the least accessible for the interaction with DOX. Using the spectrophotometric method we proved that doxorubicin may interact with some amino acids, the basic stones of all myocardial proteins, substrates for the synthesis of proteins, and products of their degradation. Some connections with these products were also found by Taetrneyer *et al.* [36].

### 2.3. Analysis of Amino Acids-Doxorubicin Interactions by IELC

**Figure 4:**
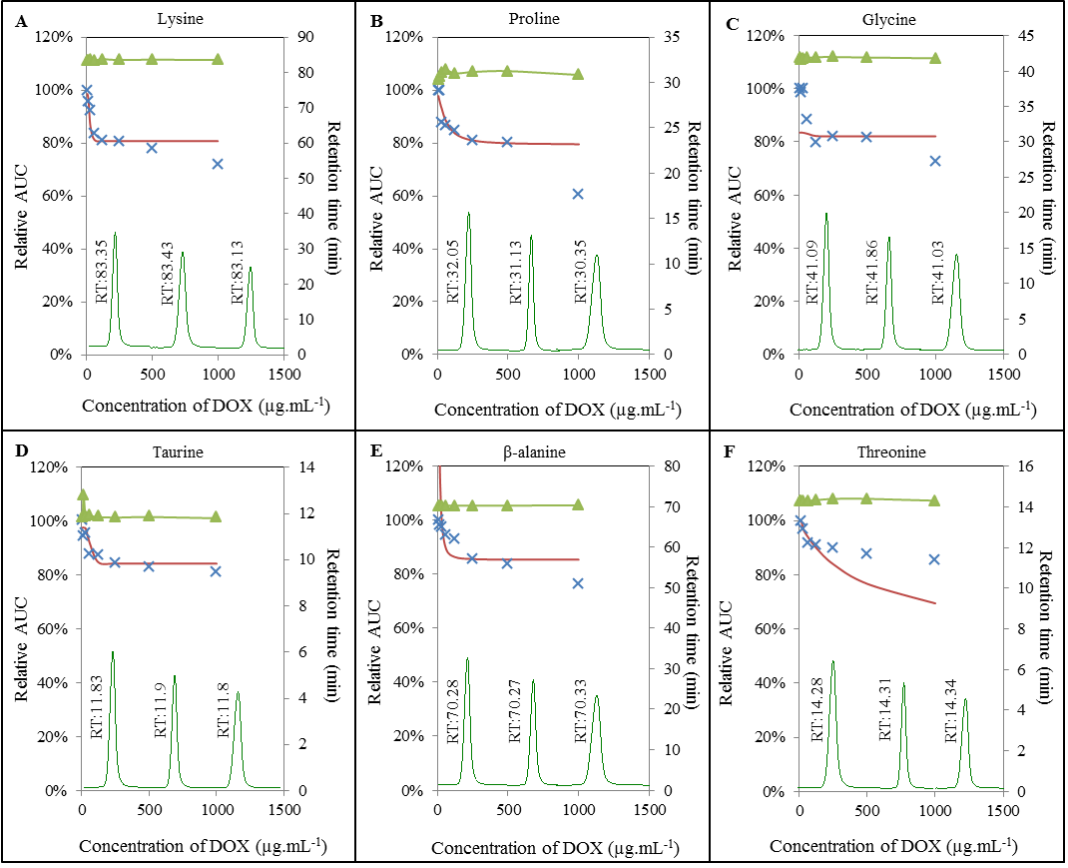
Interaction of amino acids (100 μmol mL^−1^) with doxorubicin (8; 16; 32; 64; 125; 250; 500; 1000 μg mL^−1^) monitored with IELC. DOX interaction with: (**A**) lysine; (**B**) proline; (**C**) glycine; (**D**) taurine; (**E**) β-alanine; (**F**) threonine. ▴ Expression of retention time changes (min); **×** real curve of a sample and—calculated overlay expressing amino acids breaking points.

**Figure 5:**
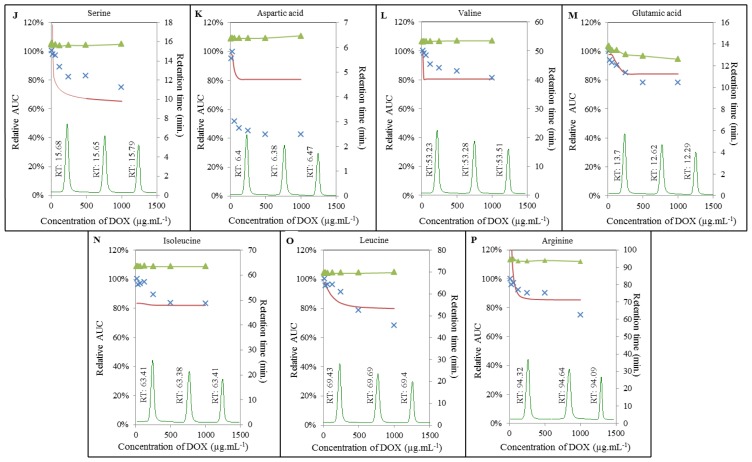
Interaction of amino acids (100 μmol mL^−1^) with doxorubicin (8; 16; 32; 62; 125; 250; 500; 1000 μg mL^−1^) monitored with IELC. DOX interaction with: (**J**) serine; (**K**) aspartic acid; (**L**) valine; (**M**) glutamic acid; (**N**) isoleucine; (**O**) leucine; (**P**) arginine. ▴ Expression of retention time changes (min); × real curve of a sample and—calculated overlay expressing amino acids breaking points.

To gain more detailed insight into the mechanisms, in terms of how doxorubicin interacts with the amino acids, we carried out IELC analysis. For this purpose, different concentrations of DOX were subjected to interaction with the constant concentration of each AA. As illustrated in Figures 4 and 5, we similarly observed apparent effect of DOX on amino acids serine, lysine, β-alanine, valine, and arginine respectively. Surprisingly, we also observed its effect on other amino acids, *i.e.*, proline, glycine, taurine, threonine, aspartic acid, glutamic acid, isoleucine, and leucine. This effect was manifested even at relatively low concentrations of doxorubicin in the range between 1 μmol mL^−1^ for serine and 84 μmol mL^−1^ for glutamic acid (see Chapter *Breaking points of amino acids*). Amino acids may act as important signaling molecules [31], especially BCAAs are effective activators of the mammalian target of rapamycin (mTOR) signalling cascade [37], which is directly involved in cardiac hypertrophy in pathways of regulation of proteosynthesis [38,39]. The main function of mTOR is the stimulation of cell growth and anabolism through increasing protein and lipid synthesis via activation of S6K (S6 kinase), 4E-BP (4E-binding protein), and SREBP (sterol response element binding protein) [40,41]. The limited availability of myocardial proteins and the potential to lose the function may induce structural alterations resulting in the formation of free radicals or in changes in antioxidant status [42–44]. Accumulation of free radicals may play a crucial role in depletion of adenosine triphosphate and subsequent opening of the non-specific mitochondrial permeability transition pores (mPTPs) [45] allowing molecules smaller than 1.5 kDa to penetrate through the mitochondrial pores and change mitochondrial membrane potential. All these effects, especially the loss of mitochondrial membrane potential, leads to the release of molecules with pro-apoptotic potential (e.g., cytochrome c) into cytosol [46,47], which results in the degradation of mitochondria, loss of myofilaments and progressive atrophy of myofibrils [48].

Additionally, we also investigated retention times of individual amino acids. Whereas most of the amino acids analyzed maintained their retention time under the influence of doxorubicin without significant changes, retention time of proline was significantly influenced by DOX in the concentration-dependent manner (Figure 4B). Retention time of proline without DOX added was established at 32.05 min. The highest concentration of doxorubicin (1000 μg mL^−1^) led to a shortening of retention time to 30.35 min. Similar changes in retention time were observed in the case of glutamic acid, where the signal was observed after 13.7 min without DOX and after 12.29 min after adding of 1000 μg mL^−1^ of doxorubicin (Figure 5M). Changes in retention time point at a possible formation of certain AA-DOX complex that has very similar properties as corresponding amino acid, but slightly shifted its retention time.

### 2.4. Effect of Doxorubicin on Breaking Points of Amino Acids

**Figure 6:**
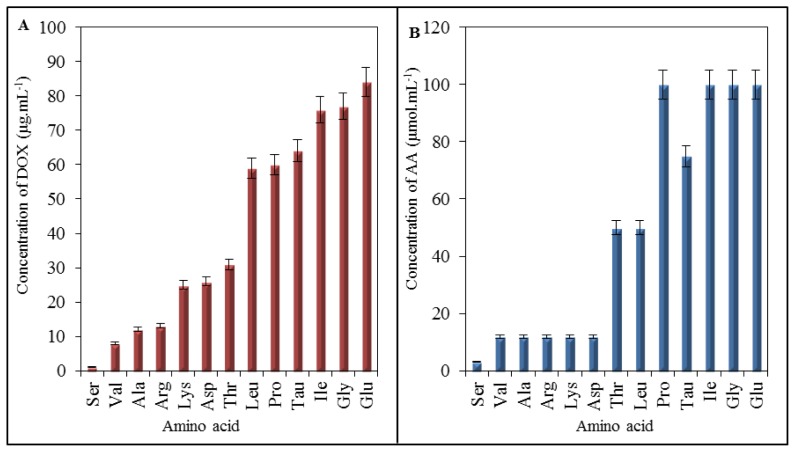
(**A**) Expression of DOX influence on amino acids via breaking points representing the lowest concentration of DOX showing a noticeable effect on amino acids. Results were obtained using IELC with postcolumn derivatization; (**B**) Expression of the effect of amino acids concentrations on DOX spectra carried out on UV/VIS spectrophotometry. There are shown amino acids concentrations, at which DOX spectra showed first observable differences.

Results of IELC analysis showed that doxorubicin interacts with amino acids, especially at low concentrations. Due to this fact, we carried out mathematical analysis of the breaking points for individual amino acids to determine the lowest concentration of DOX that causes noticeable effect on AA (Figure 6A), expressed as the lowest concentration of DOX required for formation of the complex with AA. The lowest breaking point has been determined for serine at the concentration of 1 μg mL^−1^ of doxorubicin. The highest one has been shown for glutamic acid (84 μg mL^−1^). The breaking points of all analyzed amino acids are shown in Table 1. As it is clear from Table 1, doxorubicin possesses the ability to interact with amino acids in concentrations lower than we expected. Predictive value of breaking points according to cardiotoxicity is considerable, but it is important to reveal the real amino acids composition of heart. This information may be further applied as a simple mathematical calculation revealing how much of doxorubicin has potential to influence the major amino acids—especially the most vulnerable ones. When compared with spectrophotometric analysis, serine showed the lowest breaking point, however, amino acids serine, lysine, arginine, β-alanine, and valine have very low values of breaking point; thus, these amino acids are the most accessible for interaction with DOX. Valine belongs to the important group of BCAAs that act as the activators of mTOR signalling pathway [49,50]. The depletion of these amino acids may result in the alterations in the function of mTOR with subsequent influencing of synthesis of proteins.

In addition, we assembled the summarizing output from UV/VIS spectrophotometric analysis (Figure 6B). These results are similar to the breaking points shown in Figure 6A regarding to a willingness of amino acids to interact (form a complex) with DOX. When comparing these two outputs coming from different analyses, in the case of the amino acids, which are influenced by the lowest DOX concentration simultaneously, it is valid that a low concentration of amino acid is sufficient to be influenced by the constant concentration of DOX. Serine at the concentration of 3 μmol mL^−1^ exhibited noticeable effect of DOX. At valine, lysine, arginine, β-alanine and aspartic acid sufficient concentration for the same effect was detected to 12 μmol mL^−1^. Threonine and leucine were influenced by DOX at the concentrations of 50 μmol mL^−1^, taurine at 75 μmol mL^−1^ and proline, glycine, isoleucine and glutamic acid at 100 μmol mL^−1^. The concentrations are indicative for verifying of the interaction trends and are based on the initial concentrations used for UV/VIS spectrophotometric analysis.

### 2.5. Impact of Doxorubicin on Amino Acids

**Figure 7:**
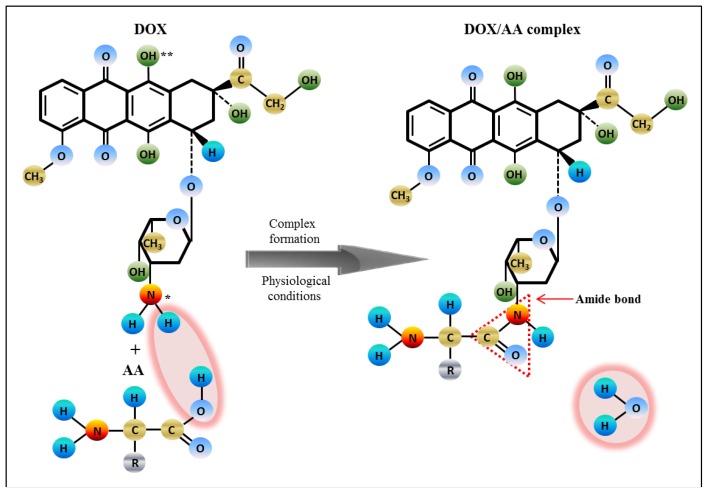
Scheme of the interaction between amino acid and doxorubicin resulting in a complex formation, where * stands for protonable functional group; ** stands for deprotonable group.

According to Manocha and Margaritis, and Yoo and Park [35,51], DOX is a positively charged amphoteric molecule, containing in its sugar moiety (daunosamine) a protonable amino group and in its aglycone part, two deprotonable phenolic groups (Figure 7). At physiological pH or in deionized distilled water, the amino group gets protonated (as NH_3_^+^) and provides positive charge to the DOX molecule [35]. Hence, it is most likely that electrostatic interactions are established between positively charged DOX and negatively charged amino acids, which results in formation of the DOX-AA complexes in dependence on binding affinity of AA. The ability of doxorubicin to form a linkage through the amide bond has been already described in several studies [52–54]. Therefore, we hypothesize that DOX-AA complexes are formed in the physiological environment found in the human body and this fact affects non-target cytotoxicity of doxorubicin.

### 2.6. Comparison of Amino Acid Profile of Myocardium before and after Application of Doxorubicin

**Figure 8:**
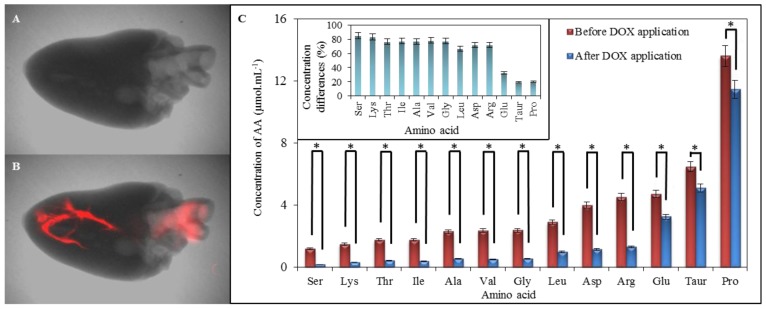
Comparison of chicken myocardium before and after application of 1000 μg mL^−1^ of doxorubicin dissolved in physiological saline solution. (**A**) Chicken cardiac muscle tissue without doxorubicin applied (X-ray image with overlaid fluorescence image); (**B**) Chicken cardiac muscle tissue with 50 μL of doxorubicin applied (X-ray image with overlaid fluorescence image). The fluorescence of doxorubicin was detected by Carestream *In Vivo* Xtreme Imaging System; (**C**) Expression of IELC results of myocardium amino acids content analysis. Both, control and heart, after application of doxorubicin were obtained as the averages from ten measurements. In inset it can be seen the percentage expression of differences between AA concentrations of amino acids in myocardium between and after application of DOX. * refer the differences between amino acid contents as statistically significant (at the *p* = 0.05 level).

We carried out scans of both hearts, untreated and treated with DOX, to confirm its presence in myocardium (Figure 8A,B). Distribution of DOX (red highlighting) mainly in heart apex and estuary of aorta is well evident (red highlighting) in Figure 8B. Compared with control, reduced levels of amino acids occurring in myocardium after exposure to doxorubicin were observed. Proline (19.94% disparity), taurine (19.03%), and glutamic acid (32.54%) were the least affected amino acids by DOX. On the other hand, serine (85.18%) and lysine (83.16%) followed by valine (78.51%) and β-alanine (77.02%) were the most affected amino acids in myocardium (Figure 8C). These data support our findings from previous measurements about increased reactivity of above mentioned amino acids and their ability to interact easily with doxorubicin.

## 3. Experimental Section

### 3.1. Chemicals and pH Measurement

Working solutions as buffers or standard solutions of amino acids and DOX were prepared daily by a diluting the stock solutions. Amino acids, DOX standards and others were purchased from Sigma Aldrich (St. Louis, MO, USA) in ACS purity, unless noted otherwise. All solutions were prepared in deionized water obtained using a reverse osmosis equipment Aqual 25 (Aqual s.r.o., Brno, Czech Republic). The deionized water was further purified by using an apparatus Direct-Q 3 UV Water Purification System equipped with an UV lamp from Millipore (Billerica, MA, USA). The resistance was established to 18 MΩ cm^−1^. The pH was measured using a pH meter WTW inoLab (Weilheim, Germany).

### 3.2. Preparation of Myocardium Samples and DOX Fluorescence Detection

For acquisition of the profiles of amino acids, ten chicken hearts were obtained (Diema s.r.o., Frydek-Mistek, Czech Republic). From each heart (*n* = 10), 10 mg of tissue was equally removed, weighed and added to 0.5 mL of 6 M HCl. Sample was subsequently subjected to digestion in a microwave reaction system Anton Paar (Anton Paar GmbH, Graz, Austria) using the following conditions: power-80, Ramp 15 min, Hold 90 min, Max 120 °C, Max pressure 25 bar, Rotor-XF-100-6. Thereafter, the digested sample was diluted 10 times with dilution buffer composed of thiodiglycol 5 mL L^−1^, citric acid 14 g L^−1^, sodium chloride 11.5 g mL^−1^ and centrifuged using a Microcentrifuge 5417R (Eppendorf AG, Hamburg, Germany) under 25,000 g at 4 °C for 10 min. The samples prepared like this were diluted with a neutralizing solution (6 M NaOH in a dilution buffer) again in ratio 1:1 and analyzed on an analyzer of amino acids (Model AAA-400, Ingos, Prague, Czech Republic). Ten chicken hearts were thereafter injected with 50 μL doxorubicin dissolved in physiological saline solution to the final concentration of 1000 μg mL^−1^. For confirmation of the presence of DOX in the myocardium, a Carestream *In Vivo* Xtreme Imaging System (Carestream Health, Inc., Rochester, NY, USA) was used to detect the fluorescence of doxorubicin after one hour-lasting exposure. Parameters were set as it follows: excitation wavelength—480 nm, emission wavelength—600 nm, exposure time—2 s, binning—2 × 2, *f*-stop—1.1, field of view—7.2 × 7.2 cm. For analysis of amino acids profile in myocardium, samples were prepared in the same way as was described above in this chapter. Analysis was then carried out at on an analyzer of amino acids (Model AAA-400, Ingos, Prague, Czech Republic) using conditions described in chapter “*Determination of content of amino acids in myocardium and analysis of interactions using IELC*”. To compare content of amino acids before and after application of DOX, the differences were expressed as percentage disparities.

### 3.3. Preparation of Amino Acid-Doxorubicin Sample for Interaction Study

The results showing the most abundant amino acids in chicken hearts were further used to monitor interactions with DOX. The stock solutions of amino acids (AA) and doxorubicin were prepared daily in the concentration of 1 mg mL^−1^ by diluting with ACS water. The final concentrations of AA and DOX were prepared by diluting with ACS water from the stock solutions unless noted otherwise. The concentrations of DOX of 8; 16; 32; 64; 125; 250; 500 and 1000 μg mL^−1^ interacting with the constant concentration of amino acids of 100 μg mL^−1^ were used for IELC evaluation of AA-DOX interactions. The concentrations of individual amino acids of 1; 2; 3; 6; 12; 25; 50 and 100 μmol mL^−1^ were used to obtain absorption spectra of AA-DOX interactions. The absorption spectra were obtained after 24 h of interaction at 25 °C with doxorubicin in concentration of 250 μg mL^−1^.

### 3.4. Determination of Content of Amino Acids in Myocardium and Analysis of Interactions Using IELC

Firstly, IELC was used to determine AA content in myocardium before and after DOX application. An ion-exchange liquid chromatography (Model AAA-400, Ingos, Prague, Czech Republic) with post column derivatization by ninhydrin and an absorbance detector in the visible light range (VIS) was used. A glass column with inner diameter of 3.7 mm and 350 mm length was filled manually with strong cation exchanger in the sodium cycle LG ANB with approximately 12 μm particles and 8% porosity. The column was tempered on the 60 °C. The double channel VIS detector with an inner cell of 5 μL volume was set to two wavelengths: 440 and 570 nm. Solution of ninhydrin was prepared in 75% *v*/*v* methylcelosolve (Ingos, Prague, Czech Republic) and in 2% *v*/*v* 4 M acetic buffer (pH 5.5). Tin chloride (SnCl_2_) was used as a reducing agent. Prepared solution of ninhydrin was stored under inert atmosphere (N_2_) in dark at 4 °C. Elution of amino acid was done by a buffer containing 10.0 g of citric acid, 5.6 g of sodium citrate, and 8.36 g of NaCl per liter of solution and pH was 3.0. Flow rate was 0.25 mL min^−1^. Reactor temperature was set on 120 °C. For dilution of samples, a dilution buffer was used (composition: thiodiglycol 5 mL L^−1^, citric acid 14 g L^−1^, sodium chloride 11.5 g mL^−1^). For monitoring of AA-DOX interactions the same parameters of analysis were used instead of the time of analysis, which depended on amino acid determined.

### 3.5. Spectrophotometric Analysis

Absorption spectra of interactions between amino acids and doxorubicin were carried out on a spectrophotometer SPECORD 210 (Analytik Jena AG, Jena, Germany) within the range from 220 to 800 nm with 1 nm step. For analysis, an UV semi-micro plastic cuvette with 1 cm optical path (Brand GMBH, Wertheim, Germany) was used. Cell compartment was thermostated to 25 °C by a thermostat Julabo (Labortechnik, Wasserburg, Germany). Absorption spectra were recorded after 24 h of the interaction and evaluated by the program WinASPECT version 2.2.7.0 (Analytik Jena AG, Jena, Germany). Finally, differential spectra of DOX-AA interactions were processed according to the formula:

(1) $${\text{spectrum}}_{\text{DOX}-\text{AA}}-{\text{spectrum}}_{\text{DOX}}$$

### 3.6. Determination of Breaking Points of Amino Acids

For the function calculating the breaking points of amino acids, following variables were defined: *a* for absorbance (*y*-axis), *l* for lowest point on *y*-axis, *u* for uppermost point on *y*-axis, *k* for slope of curve, *i* for log breakpoint position and *c* for concentration of doxorubicin. Using these variables, the relation of doxorubicin concentration and absorbance can be expressed as it follows:

(2) $$a=l-\frac{u-l}{1+10^{k(i-c)}}$$

To fit the curve, variables *l*, *u*, *k* and *i* were calculated using the least squares method. Consequently, breakpoint (*b*) was subsequently calculated according to:

(3) $$b=10^{i}$$

Macro in Microsoft Excel using a solver tool was used to create the fit of the curve and to calculate the breaking points of individual amino acids.

### 3.7. Descriptive Statistics

Mathematical analyses of the experimental data and their graphical interpretation were realized by the Microsoft Office tools (MS Excel^®^, MS Word^®^, and MS PowerPoint^®^). All results were expressed as a mean ± standard deviation (S.D.) unless noted otherwise. The detection limits (3 signal/noise, S/N) were calculated according to Long and Winefordner [55], whereas N was expressed as a standard deviation of noise determined in the signal domain unless stated otherwise. Differences with *p* < 0.05 were considered significant and were determined by using of one way ANOVA test (particularly Scheffe test), which was applied for means comparison.

## 4. Conclusions

In our study, we determined that doxorubicin induces formation of complexes with amino acids in myocardium. This fact supports the well-known knowledge about the cardiotoxicity of doxorubicin. Despite the fact that some hypotheses about the mechanism of the anthracycline-induced cardiotoxicity have been established, it has not yet been sufficiently explained. An effect of doxorubicin on amino acids may be an important factor involved in this multifactorial and very complicated process. Possible formations of complexes may play important role in the adverse effects of doxorubicin; however, this phenomenon must be further investigated. We also carried out comparison of the quantitative representation of amino acids in myocardium before and after application of doxorubicin. We observed significant reduction of levels of all amino acids in myocardium after exposure to doxorubicin. These findings lead us to knowledge that amino acids play an important role in the cardiotoxicity of doxorubicin in a dose-dependent manner. Initial amino acid composition of heart may hypothetically play an essential role in resistance of heart to doxorubicin. Nevertheless, sensitive BCAAs are very much needed for mTOR managed proteosynthesis. The solution might be found in nutrition supplements providing branched chain amino acids, and thus protecting the proper function of protein synthesis, but their effectiveness would have to be tested. It is important to reveal if DOX induced damage leads to alterations of contractility or cardiac conduction and if there is any chance how to recognize the progressive and reversible damage. From these purposes we have the future plans to carry out *in vivo* experiments, further uncovering this phenomenon. Understanding the pathophysiology of cardiac dysfunction associated with anthracyclines is important for prediction, treatment, and prevention of these adverse side effects of chemotherapy.

## Figures and Tables

**Table 1:** Overview of the breaking points expressing the lowest concentration of doxorubicin that influences noticeably the amino acids analysed. Ser—serine, Val—valine, Ala—β-alanine, Lys—lysine, Asp—aspartic acid, Thr—threonine, Leu—leucine, Pro—proline, Tau—taurine, Ile—isoleucine, Gly—glycine, Glu—glutamic acid, BP—breaking point.

Amino acid	Ser	Val	Ala	Arg	Lys	Asp	Thr	Leu	Pro	Tau	Ile	Gly	Glu
BP (μg mL^−1^ of DOX)	1	8	12	13	25	26	31	59	60	64	76	77	84

## Abbreviations

4E-BP: 4E-binding protein
AA: Amino acids
BCAAs: Branched-chain amino acids
DNR: Daunorubicin
DOX: Doxorubicin
IELC: Ion-exchange liquid chromatography
mPTPs: Mitochondrial permeability transition pores
mTOR: Mammalian target of rapamycin
ROS: Reactive oxygen species
S6K: S6 kinase
SREBP: Sterol response element binding protein.
